# Chronic Hepatitis Virus Infection Are Associated With High Risk of Gastric Cancer: A Systematic Review and Cumulative Analysis

**DOI:** 10.3389/fonc.2021.703558

**Published:** 2021-07-08

**Authors:** Yusha Yang, Zufu Jiang, Weizhou Wu, Libin Ruan, Chengyang Yu, Yuning Xi, Liling Wang, Kunpeng Wang, Jinggang Mo, Shankun Zhao

**Affiliations:** ^1^ Department of General Surgery, Taizhou Central Hospital (Taizhou University Hospital), Taizhou, China; ^2^ Department of Urology, Maoming People’s Hospital, Maoming, China; ^3^ Department of Urology, Taizhou Central Hospital (Taizhou University Hospital), Taizhou, China

**Keywords:** gastric cancer, hepatitis B virus, hepatitis C virus, cumulative analysis, risk

## Abstract

Mounting studies demonstrated both chronic hepatitis B virus (HBV) and chronic hepatitis C virus (HCV) infection might be associated not only with an increased risk of hepatocellular carcinoma but also extrahepatic malignancies, i.e., gastric cancer (GC). However, a quantitative result addressing the association between HBV/HCV infection and GC development is scarce. A systematic search to identify the eligible studies was performed in four databases, including MEDLINE, EMBASE, Cochrane Library, and the PsychINFO. The relationship between HBV/HCV infection and the risk of GC was quantified by calculating the hazard ratio (HR) with a 95% confidence interval (CI). More methodologies of this study were available in the PROSPERO (ID: CRD42021243719). Thirteen included studies involving 7,027,546 individuals (mean age, 42.6-71.9 years) were enrolled in the pooled analyses. Two articles provided the clinical data of both HBV and HCV infections. The proportion of high methodological quality studies was 76.9% (10/13). Synthetic results from 10 eligible studies of HBV showed that HBV infection was associated with a significantly higher risk of GC when compared with the healthy controls without HBV infection (pooled HR, 1.26; 95% CI, 1.08–1.47; *P* = 0.003; heterogeneity, *I^2^* = 89.3%; *P*< 0.001). In line with this finding, the combined effect derived from five included studies of HCV also supported a significant positive association between chronic HBV infection and GC development (pooled HR, 1.88; 95% CI, 1.28–2.76; *P* = 0.001; heterogeneity, *I* ^2^ = 74.7%; *P* = 0.003). In conclusion, both chronic HBV and HCV infections were related to a high risk of GC. The plausible mechanisms underlying such association might be correlated to HBV/HCV infection-induced persistent inflammation, immune dysfunction, and cirrhosis.

**Systematic Review Registration:**

PROSPERO (http://www.crd.york.ac.uk/PROSPERO), identifier (CRD42021243719).

## Introduction

Although the incidence and mortality rates are ongoing declines over the last half century in majority of countries, gastric cancer (GC) remains one of the most frequent and lethal malignancies worldwide and is responsible for over 1,000,000 new cases in 2020 and an estimated 769,000 deaths globally (1). As reported, the overall GC incidence rate is predicted to continue falling in both high-incidence and low-incidence countries (2). For example, the age-standardized incidence rate (ASR) of GC in Japan was recorded at 36 in 2010 and is anticipated to decline to 30 in 2035. With a similar trend, the ASR of GC in Australia was 5.1 in 2010 and is predicted to drop to 4.6 in 2035. However, a high incidence of GC is observed in the younger age groups (younger than 50 years) in most populations (3). This paradoxical increase of GC among younger generations is still under investigation. Till now, an increasing number of risk factors contributing to GC have been identified gradually. Chronic *Helicobacter pylori* infection is believed to be the leading cause of noncardia GC (4). Known risk factors beyond *H. pylori* infection for GC include cigarette smoking, alcohol consumption, and high consumption of unhealthy meat (i.e., processed, grilled, and barbecued meat) (1).

In addition to hepatocellular carcinoma, both chronic hepatitis B virus (HBV) and chronic hepatitis C virus (HCV) infection have gradually been recognized to associate with the development and progression of multiple extrahepatic malignancies, including colorectal cancer (5), lung cancer (6), esophageal cancer (7), and lymphoma (8). In recent years, the potential association between chronic hepatitis and GC development has been postulated and achieved special attention. In a large-sample retrospective study, Kocoglu et al. (9) found that the hepatitis B surface antigen (HBsAg) positivity rate of GC was significantly higher than the control group (5.88% *vs.* 3.3%, *P*= 0.025). In line with Kocoglu et al.’s findings, a study conducted in Iran demonstrated that HBV DNA was detected in 3.17% of GC individuals (2/63), but no HBV DNA was identified in normal individuals (0.0%, 0/21) (10). As for HCV, a nationwide population-based study indicated that HCV infection was an important risk factor for GC development (P<0.05), showing a significant difference in the cumulative incidence of GC as compared with the control group [HCV-infected sample *vs* HCV uninfected cohorts: 51/37725 (0.14%) *vs* 16/30180 (0.05%), *P*<0.05] (11). Besides, the authors also found a positive association between HCV infection and the mortality of GC (11). Although there is lack of direction of causality, a cross-sectional study developed by Baghbanian et al. (12) also indicated that HBsAg-positive patients have a higher prevalence of GC than HBsAg-negative patients (9/83, 10.8% *vs* 26/645, 4%).

Although several studies have postulated a close relationship between chronic HBV/HCV infection and GC development, some investigators demonstrated that the incidence rate of chronic hepatitis infected patients was comparable to the non-infected subjects. For example, Sundquist et al. found that chronic HBV infection was not a significant risk factor for GC (standardized incidence ratio, 0.89; 95% confidence interval, 0.35–1.85; *P*>0.05) (13). Similar to this finding, Kocoglu et al. reported that the anti-HCV positivity rate in the GC group and the control group was 1.57% and 0.84%, respectively, and no statistical difference was detected (*P*= 0.12) (9).

At present, the underlying connection between chronic HBV/HCV infection and the risk of GC is still controversial among different clinical studies. A number of more recent studies have been struggled to address this association continually (14), whereas a systematic review and meta-analysis are extremely scarce in the literature. In this study, we were intended to summarize all the evidence on the relationship between chronic hepatitis infection and risk of GC to display the risk of GC in HBV/HCV-infected individuals as compared with the healthy general population *via* a quantitative and pooled analysis, which might better facilitate the clinical understanding with this issue.

## Methods

The present systematic review and pooled analysis were performed in accordance with the guidelines of the Preferred Reporting Items for Systematic Reviews and Meta-Analyses (PRISMA). The PRISMA checklist with detail was listed in **Supplemental Table 1**. This study was registered in the PROSPERO with an access ID of CRD42021243719 (http://www.crd.york.ac.uk/PROSPERO).

### Data Sources and Search Strategy

We have systematically searched four databases, including MEDLINE (PubMed), EMBASE (OVID), the Cochrane Library, and the PsychINFO, to filter the potential studies prior to March 1, 2021. Only studies representing the English language and human participants were included. The searching strategy employed for screening the relevant studies in PubMed databases was: (“Hepatitis B, chronic”[Mesh]) OR (Chronic Hepatitis B)) OR (Hepatitis B Virus Infection, Chronic)) OR (Chronic Hepatitis B Virus Infection)) OR (HBV)) OR (Hepatitis C, Chronic)) OR (HCV)) AND (“stomach neoplasms”[Mesh]) OR (neoplasm, stomach)) OR (stomach neoplasm)) OR (Neoplasms, stomach)) OR (gastric neoplasms)) OR (gastric neoplasm)) OR (neoplasm, gastric)) OR (neoplasms, gastric)) OR (cancer of stomach)) OR (stomach cancers)) OR (gastric cancer)) OR (cancer, gastric)) OR (cancers, gastric)) OR (gastric cancers)) OR (stomach cancer)) OR (cancer, stomach)) OR (cancers, stomach)) OR (cancer of the stomach)) OR (gastric cancer, familial diffuse)). Moreover, we also reviewed the reference list to identify additional studies by a manual inspection.

### Assessments of Virus B and C Hepatitis

Patients with chronic HBV/HCV infection and GC were according to the International Classification of Diseases (ICD) using the corresponding codes and the standard of the World Health Organization (WHO). The presence/positivity of HBsAg was the most primary serum marker for identifying the cases of HBV infection. Patients with chronic HCV infection were diagnosed by anti-HCV seropositivity for 6 months or liver histology. The diagnosis of GC was confirmed by histopathological examination.

### Inclusion Criteria

Any studies reporting the incidence of GC in chronic HBV/HCV infected patients compared with a non-chronic hepatitis group were considered eligible. Besides, those studies reporting with the association between HBV/HCV infection and risk of GC by calculating with a hazard ratio (HR), odds ratios (OR), or relative risk (RR) with the 95% confidence intervals (CI) were additionally included. Moreover, studies providing sufficient data to generate the effect sizes were also included. The inclusion criteria for this systematic review and pooled analysis were in accordance with the standard of Patient, Intervention, Comparison, Outcome, and Study design (PICOS). The scientific question guiding this cumulative analysis was: Is there a positive relationship between chronic HBV/HCV infection and risk of GC? The components for the PICOS evidence were as follows: chronic HBV/HCV-infected individuals (P); diagnosis of GC or stomach cancer (I); compared with the general healthy population (C); the incidence of GC or the HR of GC (O); study designs (S).

### Exclusion Criteria

The exclusion criteria in this pooled analysis were as follows: (I) review articles, reader or editorial comments for the published studies, and case reports; (II) duplicated data or previous publications for same samples and the same scientific questions; (III) animal or *in-vitro* experiments; (IV) those studies reporting the study sample are co-infected with HBV and HCV because the present study is designed for investigating whether HBV or HCV is an independent risk factor for GC development; (V) cross-sectional studies are excluded in this meta-analysis because this kind of studies could not show the direction of causality between chronic hepatitis virus infection and the risk of gastric cancer, as it lacks the information about the temporality of associations. Based on the inclusion and exclusion criteria, the process of study selection was conducted by two authors independently. Any ambiguities were resolved by the corresponding author.

### Data Extraction

Study characteristics in every single study were extracted by two authors independently. According to a data collection form, the following information was extracted, including the first authors’ names, publication year, study area, study design, age of the participants, GC cases in the HBV/HCV group and the control group, adjusted HR with 95% CI, and variable adjustment in each included study.

### Quality Assessment

The methodological quality of the cohort studies and case-control studies was evaluated by the Newcastle–Ottawa Scale (NOS), in which low quality, moderate quality, and high quality were based on the scores of 0–3, 4–6, and 7–9, respectively.

### Statistical Methodology

The pooled analysis was conducted by using STATA version 13.0 software for Windows (Stata Corp LP, College Station, Texas, USA). The overall HR with 95% CI synthesized by all the included studies was used to quantitatively evaluate the strength of the relationship between HBV/HCV infection and the risk of GC. Statistical significance was assumed at a two-tailed *P* values < 0.05. *I* ^2^ statistics and the Cochrane *Q* statistic were used for the heterogeneity test. *I* ^2^ > 50% was regarded as substantial heterogeneity, whereas the *P*-value of the *Q* test < 0.10 was considered to be statistically significant. On account of a high likelihood of between-study variance for differences in a designed study and the demographic characteristics, a random-effects model rather than a fixed-effects model was used in the current study. Subgroup analyses on different important factors were performed to better illustrate the association between HBV/HCV infection and risk of GC and further explore the origin of heterogeneity. Additionally, sensitivity analyses were applied to identify the potential origin of the between-study heterogeneity. The funnel plot, Begg’s rank-correlation test, and Egger’s regression asymmetry test were used to evaluate the publication bias.

## Results

### Literature Search

**Figure 1:**
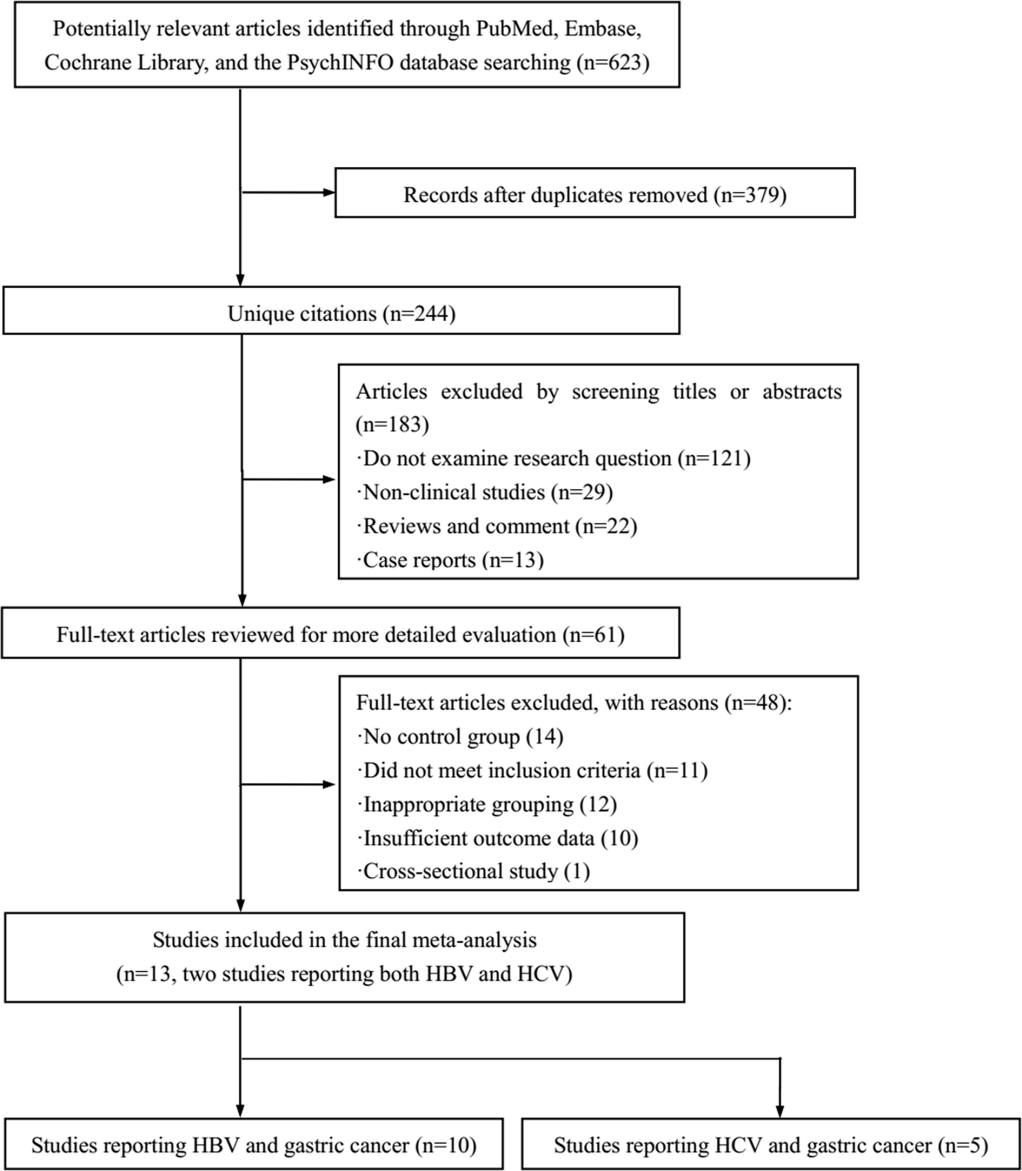
Flow chart of study selection.

The selection process for screening the eligible articles is shown in **Figure 1**. A total of 623 articles were identified during the initial searching in the four databases. After excluding duplicates, non-clinical studies, studies out of the research question, review articles, comments, and case reports, 562 publications were eliminated, and the remaining 61 potentially related articles were retrieved for the full-text review. Among these studies, 14 studies were eliminated for lacking control group, 11 studies did not meet the inclusion criteria, 12 studies for inappropriate grouping; 10 studies were excluded due to insufficient outcome data, and 1 study was eliminated for its study design was cross-sectional. Finally, 13 studies (11, 13, 15–25) met the aforementioned inclusion criteria and thus were included in this pooled analysis. Of which, two included studies (16, 22) have provided the data of both HBV and HCV. As a result, 10 included studies reporting HBV (13, 15–23) and five included studies (11, 16, 22, 24, 25) reporting HCV were used for pooling the overall HR.

### Study Characteristics

The publication years of the 13 eligible studies ranged from 2014 to 2020. The mean age of the participants ranged from 42.6 ± 12.6 to 71.9 ± 14.59 years. For the study geographical area, 10 studies were conducted in Asia, 2 studies in the United States, and 1 study in Europe. The study design of the eligible studies included cohort and case-control studies. The sample size among these included ranged from 1,160 to 5,332,903, involving a total of 7,027,546 individuals. Among the 13 included studies, two studies (Kamiza et al. and Hong et al.) (16, 22) providing the clinical information of both HBV and HCV. In An et al.’s study (18), the adjusted HR contained both female and male subjects independently. In Lu et al.’s study (19), the adjusted HR was divided into different age groups, including 0–39, 40–59, and ≥ 60 years old. In Song et al.’s study (21), there were three adjusted HR from three cohorts based on the different study areas of China, including Beijing city, Qingdao city, and Guangzhou city. The characteristics of the 15 eligible studies were summarized in **Table 1**.

**Table 1:** Characteristics of the included studies.

Study	Study area	Study design	Mean age (years)	Study groupcase/total	Control groupcase/total	Adjusted HR with 95% CI	Variable adjustment
***Twelve included studies reporting the association between HBV and risk of GC:***							
Sundquist et al. (13)	Sweden	Cohort	A broad age	NA/10,197	NA	0.89 (0.35-1.85)	Age, occupation (socioeconomic status)
Wei et al. (15)	China	Case– control	A broad age	NA/170	NA/990	1.49 (1.06-2.10)	Age, sex, year of diagnosis
Kamiza et al. (16)	Chinese Taipei	Cohort	A broad age	32/NA	132/NA	1.42 (1.00-2.01)	Sex, age, geographical region, occupation, level of urbanization, monthly income, the presence of comorbidities, and number of outpatient visits
Wei et al. (17)	China	Cohort	Median age: S: 50; C: 54	NA/8,418	NA	1.24 (1.06-1.45)	Age, sex, year of diagnosis, smoking, drinking, and family history of cancer
An et al. (18)	Korea	Case– control	M: 56-73; F: 47-66	M: 519/NA; F: 184/NA	NA	M: 1.03 (0.90-1.17); F: 0.98 (0.79-1.21)	Age, hypertension, diabetes, body mass index, alcohol consumption, smoking status, and cholesterol level
Lu et al. (19)	China	Case– control	S: 55.0 ± 12.7; C: 58.9 ± 12.9	961/NA	5,986/NA	0-39 years old: 1.763 (1.424–2.169); 40-59 years old: 1.284 (1.12–1.472); ≥ 60 years old: 0.631 (0.549–0.725)	Age
Mahale et al. (20)	USA	Cohort	NA	11039/NA	NA	1.19 (1.03-1.37)	Age, sex, race, year of cancer diagnosis, smoking status
Song et al. (21)	China	Three cohorts	Cohort 1: 30-79; Cohort 2: 30-70; Cohort 3: >30	Cohort 1: 78/15,355; Cohort 2: 19/3,539; Cohort 3: NA	Cohort 1: 2079/481377; Cohort 2: 109/33797; Cohort 3: NA	Cohort 1: 1.41 (1.11-1.8); Cohort 2: 2.02 (1.24-3.29); Cohort 3: 1.76 (1.04-2.98)	Age, sex, region, educational level, household income, marital status, smoking status, alcohol consumption, family cancer history, BMI
Hong et al. (22)	Korea	Cohort	42.6 ± 12.6	NA/26,665	NA/500,680	1.39 (1.22-1.58)	Sex, BMI, smoking, drinking, income percentile, residential area, comorbidities
Tian et al. (23)	China	Case– control	S: 57.7 ± 13.9; C: 55.8 ± 17.4	609/NA	7284/NA	1.46 (1.3-1.65)	Age and gender
***Five included studies reporting the association between HCV and risk of GC:***							
Kamiza et al. (16)	Chinese Taipei	Cohort	A broad age	37/NA	122/NA	1.78 (1.28-2.46)	Sex, age, geographical region, occupation, level of urbanization, monthly income, comorbidities, number of outpatient visits
Chen et al. (11)	China	Cohort	S: 49.73 ± 11.25; C: 49.12 ± 48.98	51/37,725	16/30,180	2.55 (1.455-4.471)	Age, sex, and some comorbidities
Hong et al. (22)	Korea	Cohort	42.6 ± 12.6	NA/7,251	NA/500,680	1.03 (0.75-1.42)	Sex, BMI, smoking, drinking, income percentile, residential area, comorbidities
Huang et al. (24)	Chinese Taipei	Cohort	S: 54.3 ± 11.4	NA/10,714	NA	3.41 (1.42-8.19)	Age and sex
Nyberg et al. (25)	USA	Cohort	S: 59.6 ± 8.4; C: 71.9 ± 14.59	185/35,712	NA/5,297,191	2.195 (1.521-3.165)	Age, gender, race, smoking, and cirrhosis

S, study group: patients with HBV or HCV infection; C, control group: the healthy general population without HBV/HCV infection; NA, not available; GC, Gastric cancer; HR, Hazard ratio; CI, Confidence interval; BMI, Body Mass Index.

### Study Quality

Based on the NOS, three included studies (13, 19, 24) were considered to be moderate quality and 10 included studies (11, 15–18, 20–23, 25) were high quality. The proportion of high methodological quality study was 76.9% (10/13). The detail-specific scoring of the study quality was shown in **Supplementary Table 2**.

### Pooled Analysis

**Figure 2:**
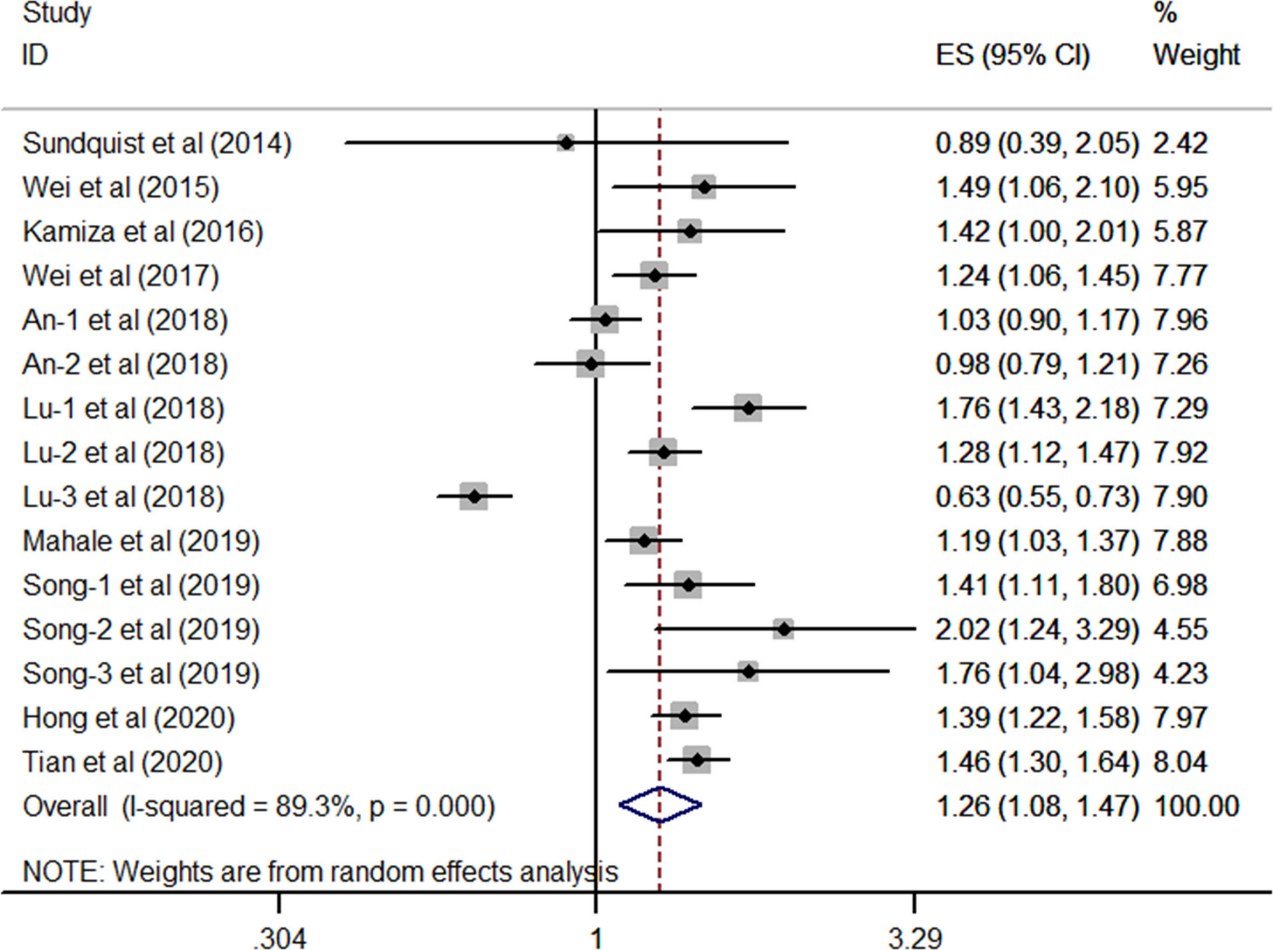
Forest plots of the pooled analysis of the included studies on the association between HBV and risk of gastric cancer.

As shown in **Figure 2**, after multivariable adjustment, the pooled effect from 10 included studies reporting HBV supported a significant positive relationship between chronic HBV infection and high risk of GC (overall synthetic HR derived from 15 single HR, 1.26; 95% CI, 1.08–1.47; *P*=0.003) by using a random-effects model. Statistical heterogeneity was identified during this meta-analysis (*I^2^* = 89.3%, *P*< 0.001).

In the present study, we have conducted subgroup analyses to detect the source of heterogeneity in the relationship between HBV infection and the risk of GC. Notably, the disease duration of HBV infection, antiviral therapy, gender, and mean age among different studies might be the confounding factors. However, only limited included studies have provided detail information on the above factors, so we could not perform a subgroup analysis on these confounding factors.

**Figure 3:**
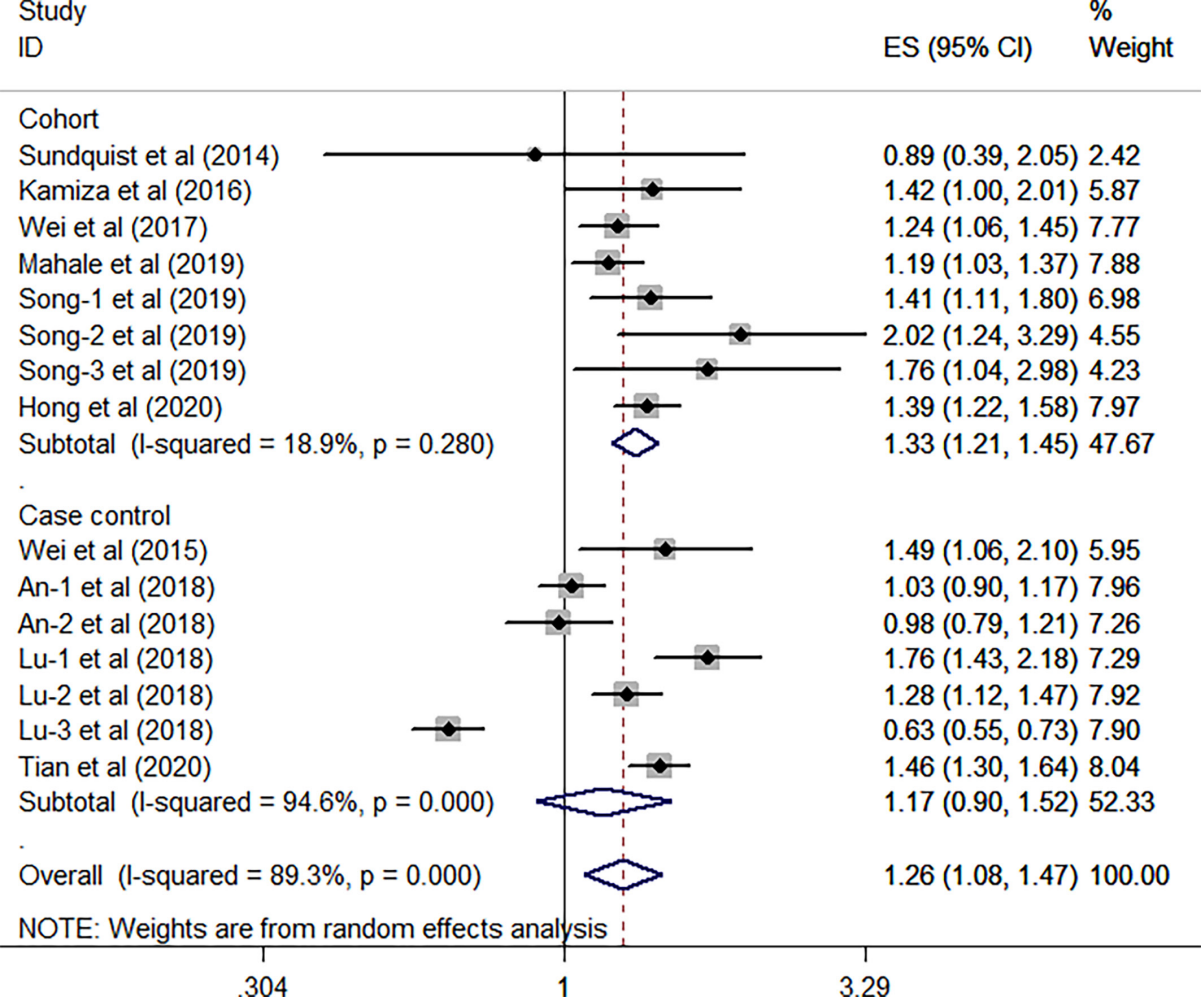
Forest plots of the subgroup analysis (study design) on the association between HBV and gastric cancer.

**Figure 4:**
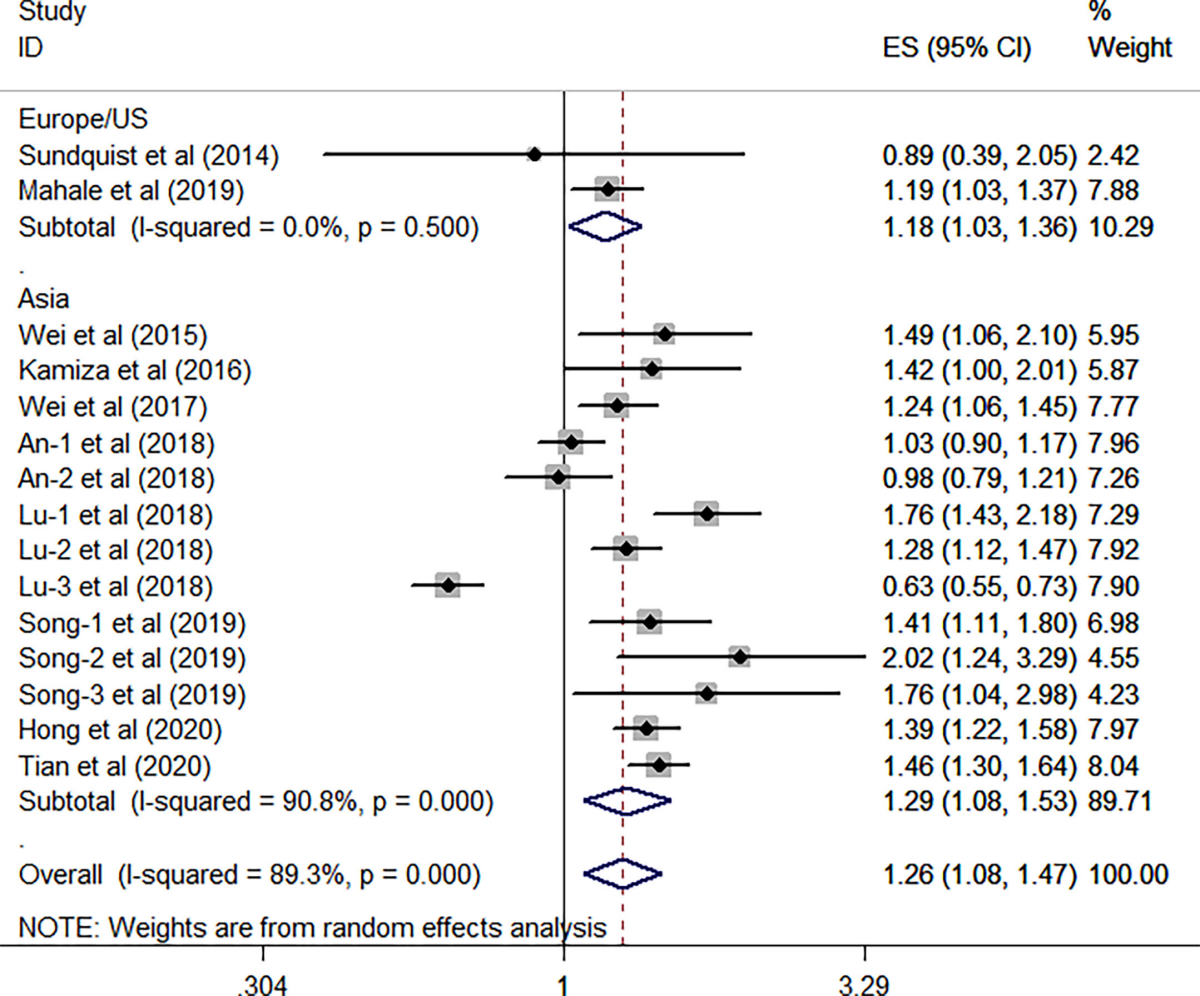
Forest plots of the subgroup analysis (research areas) on the association between HBV and gastric cancer.

All the 10 eligible studies reporting the association between HBV infection and GC have provided the study design and the research area; thus, a subgroup analysis was conducted on the two factors. As shown in **Figure 3**, results from the subgroup analysis on the study design showed that the relationship between HBV infection and high risk of GC existed in the eight cohort studies (HR, 1.33; 95% CI, 1.21–1.45; *P <*0.001), and no significant heterogeneity was identified (*I* ^2^ = 18.9%, *P* = 0.28). However, such association was not found in the seven case-control studies (HR, 1.17; 95% CI, 0.90–1.52; *P* =0.247), and the substantial heterogeneity was detected (*I* ^2^ = 94.6%, *P <*0.001). When focused on the geographical area, this subgroup analysis revealed that the positive association between HBV infection and the risk of GC was existed either in the Europe/US or the Asian countries (all *P*> 0.05). Furthermore, we have found that such association is stronger in the Asian countries (HR, 1.29; 95% CI, 1.08–1.53; *P* = 0.004; *I* ^2^ = 90.8%; *P* < 0.001) than the Europe/US (HR, 1.18; 95% CI, 1.03–1.36; *P* = 0.021; *I* ^2^ = 0.0%, *P* = 0.5) (**Figure 4**).

**Figure 5:**
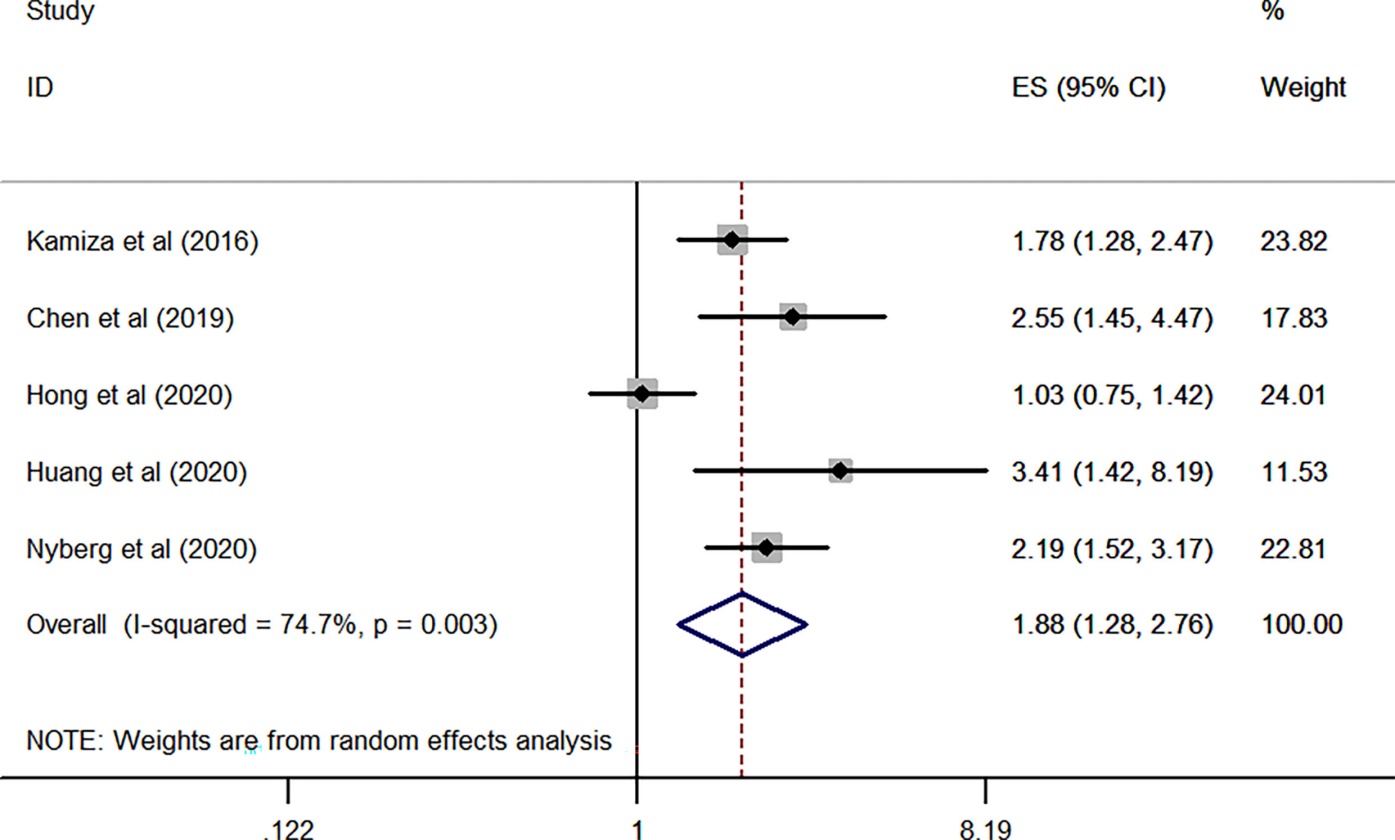
Forest plots of the pooled analysis of the included studies on the association between HCV and risk of gastric cancer.

As for HCV, the combined results from FIVE independent HR of each included study revealed that HCV infected patients was significantly associated with an increased risk of GC when compared WITH the general population without HCV infection (overall HR of five studies, 1.88; 95% CI, 1.28–2.76; *P* = 0.001; heterogeneity, *I* ^2^ = 74.7%; *P* = 0.003) (**Figure 5**).

Based on the above results, the stratified analysis revealed that patients with HCV infection exhibited an increased odds of GC as compared to those with HBV infection (adjusted HR, 1.88 *vs* 1.26).

### Sensitivity Analysis

**Figure 6:**
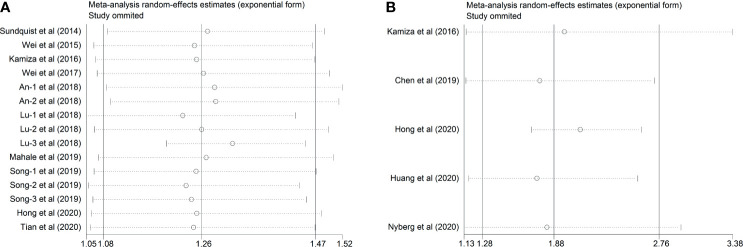
Sensitivity analysis after each study was excluded by turns. **(A)** Studies of HBV and GC **(B)** Studies of HCV and GC.

In this study, sensitivity analysis from both of the studies of HBV and HCV was conducted to assess the influence of individual study on the newly generated overall HR. As listed in **Table 2** and **Figure 6A**, there was no substantial change in the overall combined HR for those studies reporting the HBV cases, which ranged from 1.23 (95% CI, 1.053–1.437; *P*= 0.009) to 1.49 (95% CI, 1.059–2.097; *P*= 0.002) after excluding any of the studies. Besides, similar heterogeneity was observed after each exclusion, the *I* ^2^ ranged from 67.4% to 90.1% (all *P*< 0.001). The abovementioned results indicated that no single study dominated the pooled HR and heterogeneity in those studies reporting HBV.

**Table 2:** Sensitivity analysis after each study was excluded by turns in those studies reporting HBV and risk of gastric cancer.

Study omitted	RR (95% CI) for remainders	Heterogeneity	
		*I* ^2^	*P*
Sundquist et al. (13)	1.490 (1.059–2.097) *P*=0.002	90.1%	<0.001
Wei et al. (15)	1.251 (1.067–1.467) *P*=0.006	90.0%	<0.001
Kamiza et al. (16)	1.255 (1.070–1.472) *P*=0.005	90.0%	<0.001
Wei et al. (17)	1.268 (1.073–1.498) *P*=0.005	90.1%	<0.001
An-1 et al. (18)	1.288 (1.090–1.522) *P*=0.003	89.6%	<0.001
An-2 et al. (18)	1.290 (1.098–1.516) *P*=0.002	89.8%	<0.001
Lu-1 et al. (19)	1.230 (1.053–1.437) *P*=0.009	88.9%	<0.001
Lu-2 et al. (19)	1.265 (1.068–1.497) *P*=0.006	90.0%	<0.001
Lu-3 et al. (19)	1.321 (1.200–1.454) *P*<0.001	67.4%	<0.001
Mahale et al. (20)	1.273 (1.075–1.506) *P*=0.005	90.1%	<0.001
Song-1 et al. (21)	1.254 (1.067–1.474) *P*=0.006	89.9%	<0.001
Song-2 et al. (21)	1.236 (1.058–1.444) *P*=0.008	89.7%	<0.001
Song-3 et al. (21)	1.245 (1.065–1.456) *P*=0.006	89.9%	<0.001
Hong et al. (22)	1.255 (1.063–1.483) *P*=0.007	89.6%	<0.001
Tian et al. (23)	1.249 (1.060–1.472) *P*=0.008	89.0%	<0.001

HR, hazard ratio; CI, confidence interval.

As for the five included studies reporting HCV, sensitivity analysis demonstrated that there was no remarkable variation in the pooled HR after omitting any one of the included studies the new HR ranged from 1.739 (95% CI, 1.169–2.586; *P*= 0.006) to 2.103 (95% CI, 1.693–2.612; *P*< 0.001). Intriguingly, the substantial heterogeneity disappeared after omitting the study conducted by Hong et al.’s study (22) (*I^2^* = 0.0%, *P*= 0.444), suggesting that this study was the origin of the substantial heterogeneity of the combined effect (**Table 3** and **Figure 6B**).

**Table 3:** Sensitivity analysis after each study was excluded by turns in those studies reporting HCV and risk of gastric cancer.

Study omitted	RR (95% CI) for remainders	Heterogeneity	
		*I* ^2^	*P*
Kamiza et al. (16)	1.968 (1.146–1.380) *P*=0.014	80.8%	0.001
Chen et al. (11)	1.766 (1.145–2.721) *P*=0.01	77.7%	0.004
Hong et al. (22)	2.103 (1.693–2.612) *P*<0.001	0.0%	0.444
Huang et al. (24)	1.739 (1.169–2.586) *P*=0.006	77.3%	0.004
Nyberg et al. (25)	1.824 (1.129–2.945) *P*=0.014	77.1%	0.004

HR, hazard ratio; CI, confidence interval.

### Publication Bias

**Figure 7:**
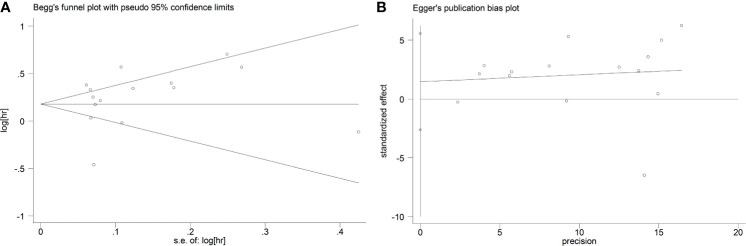
Begg’s **(A)** and Egger’s **(B)** tests to detect publication bias in studies of HBV and GC.

**Figure 8:**
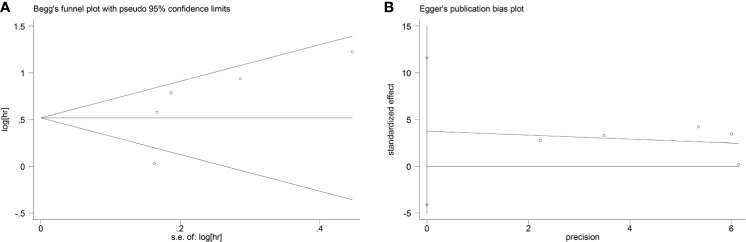
Begg’s **(A)** and Egger’s **(B)** tests to detect publication bias in studies of HCV and GC.

As displayed in **Figure 7**, the funnel plots indicated that no significant publication bias was found among the included studies reporting the HBV cases (Begg’s, *P* > |z| = 1.000; Egger, *P* > |t| = 0.455; 95% CI, −2.64 to 5.55). Similar to this finding, the funnel plots from five eligible studies reporting HCV infection cases also demonstrated that no significant publication bias existed in the combined HR (Begg’s, *P* > |z| = 0.221; Egger, *P* > |t| = 0.228; 95% CI, −4.13 to 11.61) (**Figure 8**).

## Discussion

Mounting studies have confirmed a significant positive association between virus infection and the development of various malignancies, i.e., Epstein-Barr (EB) virus in nasopharyngeal carcinoma (26), human papillomavirus (HPV) in cervical cancer (27), and human cytomegalovirus (HCMV) in glioblastoma (28). Type B viral hepatitis is one of the most common communicable diseases afflicting humans. It was estimated that 257 million persons worldwide have suffered from chronic HBV infection (29). Chronic HBV infection is the predominant cause of hepatocellular carcinoma (HCC). Besides, increasing evidence has postulated a potential relationship between HBV infection and risk of extrahepatic cancer, i.e., colorectal cancer (5), esophageal cancer (7), and pancreatic cancer (30). Moreover, recent studies also indicated that HBV infection might increase the risk of GC (31). Type C viral hepatitis is also one of the common infectious diseases originating from the liver. As reported, chronic HCV infection can induce the development of both HCC and extrahepatic malignancies (i.e., GC) because of the persistent inflammation caused by HCV (32). Over the past few decades, a growing number of studies have investigated the relationship between HBV/HCV infection and the tumorigenesis of GC but provided inconsistent results, and a quantified result is still scarce. Based on the pooled data from 12 relevant studies exploring the association between HBV infection and GC, the combined effect revealed that that HBV infected patients (HBsAg positive) were at 1.26-fold higher risk of GC than the general population without HBV infection with a statistical significance (RR, 1.26; 95% CI, 1.08−1.47; *P* = 0.003). This result was consistent with two previous meta-analyses related to this topic, which also supported a significant positive relationship between HBV infection and GC development (31, 33). Up to date, there is no meta-analysis yet that has been published on the issue of the association between HCV infection and the risk of GC. According to the present study with a pooled analysis of five included studies, the risk of GC in HCV-infected patients was increased by 88% compared with those without HCV infection (combined HR, 1.88; 95% CI, 1.28−2.76; *P* = 0.001). In the present study, sensitivity analyses showed that the quantification of the risk for GC in patients with either HBV or HCV remained prominently higher in all of the included studies (the HR in those studies reporting HBV ranged from 1.23 to 1.49; the HR in five studies reporting HCV ranged from 1.739 to 2.103), indicating that our findings were robust. In this study, all the HRs were adjusted for the common confounders, including the known risk factors for GC (e.g., *Helicobacter pylori* infection, chronic gastritis, and family history of GC) and some other factors, such as age, sex, geographical region, occupation, and comorbidities. Based on the abovementioned evidence, a significant positive relationship between HBV/HCV infection and GC development was verified. Also, HBV/HCV infection might be an independent risk factor for the tumorigenesis of GC.

In the subgroup analysis of the study design, studies with cohort design have verified the positive association between HBV infection and the high prevalence of GC but such association was eliminated in those studies with a case-control design. According to the epidemiologic investigation, a cohort is defined as studies that sample participants on the basis of exposure and assess the outcome during follow-up. In a cohort study, patients are sampled on the basis of exposure and are followed up over time, and the occurrence of outcomes is assessed. Case-control studies, the common analytical epidemiological studies, are the only practical approach for identifying risk factors for some diseases. Case-control studies lack temporality because they are retrospective designs. Therefore, the methodological quality and the level of evidence are higher in cohort studies as compared with case-control studies. Given this inconsistent outcome, further well-signed cohort studies are still warranted to validate the evidence of HBV infection predisposing to the development of GC. In the subgroup analysis of different research areas, pooled results from studies conducted in the Asian countries had a higher prevalence of GC when compared with the Europe/US. This phenomenon may be attributable to the prevalence and the disease severity of HBV infection, which are higher in Asian countries than those in Europe and the United States. However, few studies have been conducted to investigate the prevalence of GC in HBV infection patients in both Asian and European countries. Therefore, the etiology underlying this observation is still unknown.

The pathophysiological mechanisms of HBV/HCV-associated GC development remain elusive. The underlying mechanisms that existed in the association between HBV/HCV infection and the development of GC might be correlated with multiple etiologies, including chronic inflammation, systemic impairment of immune function, liver cirrhosis, and the direct effects of HBV/HCV proteins on oncogenesis and/or on tumor suppression genes (34, 35).

HBV is one of the most common hepatotropic viruses, which replicate in the hepatocytes. Viral oncogenic hepatitis B virus X proteins (HBX) have been confirmed to play a key role in the development of multiple cancers. Intriguingly, however, some investigators also found that HBX was significantly higher in GC cells than in healthy parts of the specimens (21). In addition, viral antigens and genomes have been identified in extra-hepatic tissues. Therefore, HBV might be not only harbored in the hepatocytes but also in the extrahepatic cells and thus induce local chronic inflammation of this organ subsequently, such as in the stomach. It was suggested that chronic inflammation is a crucial component of cancer progression (36). Local persistent inflammation caused by the HBV might promote the cancerous transformation of gastric epithelial cells. Chronic inflammation might also lead to genetic instability in cells and further elevate the genetic and epigenetic alterations. Supported by this evidence, Cui et al. found that HBV infection in epithelium could induce cellular atypia and generate a prominent lymphocytes’ infiltration in lamina propria, indicating the persistence of long-term inflammation context in the gastric tissues (31). Long-term chronic HBV infection might be concurrent with damage of gastric mucosa epithelial cells, thus promoting the development of GC (15, 37).

It is well known that immune dysregulation facilitates the development of cancer (38). The impaired immune system also plays a vital role in the development of HBV infection-associated GC. Infectious agents increase the risk of GC, which might be *via* the integration of their genomes into the host’s chromosomes or immune system inhibition (10). Song et al. (21) speculated the potential link between HBV infection and GC might be owing to the specific influence of the virus on the immune system or more vulnerable to a malicious attack on the gastric histiocytes. Chronic immune suppression may also participate in this pathological process. In China, a majority of HBV carriers infected HBV during their childhood, leading to long-term immune suppression, which may contribute to a higher incidence of GC. In contrast, those individuals never exposed to HBV or with antibody-positive hepatitis B surface (anti-HBs+) are immune to HBV, having a decreased risk of GC (23). In addition to the immune mechanisms, some relevant viral proteins and the intracellular cascades pathways function may also play role in the action of HBV infection-induced GC. The integration and mutation of the viral genome into the host cellular DNA contribute to the altered expression of some cellular genes, which can induce direct insertional mutagenesis of various cancer-related genes and thus lead to malignant transformation (17). The stomach is adjacent to the liver and thus may be infected by HBV. Several investigators observed that HBsAg presented in the gastric epithelial cells (15, 39), suggesting that HBV directly increased the risk of GC.

In addition to chronic inflammation and immune dysregulation, liver cirrhosis may also play an important role in the carcinogenesis of GC. According to the WHO, about 20% of the chronically HBV-infected patients will develop cirrhosis (40). HBsAg is proven to be an independent risk factor for liver cirrhosis (41), whereas cirrhosis has been reported to be a risk factor for GC (42). It was suggested that liver cirrhosis might cause hypoxia, which was not only a risk factor for GC but also a poor prognosis for patients with GC (43, 44). Besides, a meta-analysis that included 21 eligible studies showed that there was a significantly high prevalence of *H. pylori* infection in patients with liver cirrhosis (45). It is known that there is a close association between *H. pylori* infection and the development of GC (46). Therefore, there might be a chain reaction effect of “HBV–liver cirrhosis–*H. pylori* infection–GC”. Of note, although liver cirrhosis may play a role in increasing the risk of GC in HBV-infected people, the association between HBV infection and GC persisted among non-cirrhotic subjects (20). Therefore, more studies are still warranted to better illustrate this issue.

The positive association between HCV infection and the risk of GC shares many common pathogenetic risk factors, including chronic inflammation, immune dysfunction, cirrhosis, and the altered expression of the related oncogenes. It was reported that HCV acted as an indirect carcinogen for GC by promoting and maintaining a state of chronic inflammation in infected sites (47). HCV infection-induced chronic inflammation may lead to the progressive rearrangement of gastric tissue structure and thus promote the cancerous transformation (48). Valli De Re et al. (49) found that HCV infection might induce aberrant expression of MHC-I peptides, favoring the NK-mediated cell lysis and producing cytokines and chemokines, resulting in inflammation in the stomach. HCV might be also associated with the progressive development of a tissue necroinflammatory process in the infected-organs (i.e., the stomach), which might evolve toward malignant transformation (48). Immune and/or endocrine dysregulation after HCV infection might partially attribute to the increased risk of GC (11, 50). A previous study (49) demonstrated that a few proteins involved in oxidative stress responses were over-expressed in HCV-positive gastric tissue. The relationship between oxidative stress and cancer has been studied extensively (51), thus it is tempting to speculate that HCV may induce GC development and that oxidative stress involves in this action. Similar to one of the pathological mechanisms laid in HBV and GC, Nyberg et al. (25) found a significant relationship between the presence of cirrhosis and the increased GC risk *via* the multivariable analyses and suggested that the underlying mechanisms might correlate to the general immune response in cirrhosis. The direct action of some oncogenic viral proteins induced by HCV has also been implicated in tumorigenesis of various extrahepatic cancers (52), whereas whether this theory exists in the development of GC is still under investigation. Based on this evidence, further understanding of the possible mechanism laid in HBV infection and the risk of GC might provide a novel strategy to prevent the tumorigenesis and progression of GC.

In this systematic review and meta-analysis, we have not only addressed the association between HBV infection and GC development but also quantified the association between HCV infection and the risk of GC. However, an inherent limitation within this study should be noted during the interpretation in clinical practice. Statistical heterogeneity has been observed in this study, for either HBV or HCV pooled analysis. Inconsistency of study geographical area, study design, sample size, mean age, HBV/HCV-infected duration and severity, and the different variable adjustments could be partly responsible for such heterogeneity.

In conclusion, the present study demonstrated that both HBV and HCV infection was associated with a significantly higher risk of GC. HCV infection had a higher odds of GC development when compared to HBV infection. A few plausible mechanisms might explain the causality of HBV/HCV infection and the increased risk of GC, i.e., persistent inflammation, immune dysfunction, and cirrhosis. However, such causal association remains inconclusive among studies, thus further investigations are still required.

## Data Availability Statement

The original contributions presented in the study are included in the article/**Supplementary Material**. Further inquiries can be directed to the corresponding author.

## Author Contributions

YY, ZJ, and WW contributed to conceive and design the study. LR and CY performed the systematic searching. YX and KW extracted the data. SZ and JM wrote the manuscript. YY and SZ supervised the manuscript. All authors contributed to the article and approved the submitted version.

## Funding

This work was supported by the grants from Clinical Research Funding of Zhejiang Medical Association (ID: 2019ZYC-A182), Science and Technology Planning Project of Taizhou City, Zhejiang Province (ID: 1902ky37 and 20ywb40), the Scientific Research Project of Taizhou University (ID: 2017PY047), and the High-level Hospital Construction Research Project of Maoming People’s Hospital.

## Conflict of Interest

The authors declare that the research was conducted in the absence of any commercial or financial relationships that could be construed as a potential conflict of interest.

## Abbreviations

ASR, age-standardized incidence rate; CI, confidence interval; GC, gastric cancer; HBV, hepatitis B virus; HCV, hepatitis C virus; HR, hazard ratio; NOS, Newcastle–Ottawa scale.
