# Supplementary material for: Chronic Hepatitis Virus Infection Are Associated With High Risk of Gastric Cancer: A Systematic Review and Cumulative Analysis
Source: Front Oncol. 2021 Jul 8;11:703558. doi: 10.3389/fonc.2021.703558 (PMC8297975; doi:10.3389/fonc.2021.703558)
Supplement: Supplementary file 2 [file Table_2.doc]

| Study | Selection | | | | Comparability | | Exposure/Outcome | | | Total scores |
| --- | --- | --- | --- | --- | --- | --- | --- | --- | --- | --- |
|  | 1 | 2 | 3 | 4 | 5 | 6 | 7 | 8 | 9 |  |
| Sundquist et al (13) (2014) | Yes | Yes | Yes | Yes | Yes | No | No | No | No | 5 |
| Wei et al (15) (2015) | Yes | Yes | Yes | Yes | Yes | Yes | Yes | No | No | 7 |
| Kamiza et al (16) (2016) | Yes | Yes | Yes | Yes | Yes | No | Yes | Yes | Yes | 8 |
| Wei et al (17) (2017) | Yes | Yes | Yes | Yes | Yes | Yes | Yes | No | Yes | 8 |
| An et al (18) (2018) | Yes | Yes | Yes | Yes | Yes | Yes | No | Yes | No | 7 |
| Lu et al (19) (2018) | Yes | Yes | Yes | Yes | Yes | No | Yes | No | No | 6 |
| Mahale et al (20) (2019) | Yes | Yes | Yes | Yes | Yes | No | Yes | Yes | Yes | 8 |
| Song et al (21) (2019) | Yes | Yes | Yes | Yes | Yes | No | Yes | Yes | Yes | 8 |
| Hong et al (22) (2020) | Yes | Yes | Yes | Yes | Yes | Yes | Yes | No | No | 7 |
| Tian et al (23) (2020) | Yes | Yes | Yes | Yes | Yes | Yes | Yes | No | Yes | 8 |
| Chen et al (11) (2019) | Yes | Yes | Yes | Yes | Yes | No | Yes | No | Yes | 7 |
| Huang et al (24) (2020) | Yes | Yes | Yes | Yes | No | Yes | No | Yes | No | 6 |
| Nyberg et al (25) (2020) | Yes | Yes | Yes | Yes | Yes | No | Yes | Yes | No | 7 |

Supplementary Table 2. Newcastle-Ottawa Scale assessment of the quality of the case-control and cohort studies.

NOTE: 1. indicates that the exposed cohort was representative of the population; 2. Indicates that the non-exposed cohort was drawn from the same population; 3. Indicates that the exposure ascertainment was from secure records or a structured interview; 4. Indicates that outcome of interest was not present at start of study; 5. Indicates that the cohorts were comparable for age and sex; 6. Indicates that the cohorts were comparable on all additional factor(s) reported; 7. Indicates that outcome was assessed from a secure record; 8. Indicates that follow-up was long enough for outcomes to occur; 9. Indicates that follow-up was complete.
